# Brain injury, neuroinflammation and Alzheimer's disease

**DOI:** 10.3389/fnagi.2013.00026

**Published:** 2013-07-11

**Authors:** Joshua J. Breunig, Marie-Victoire Guillot-Sestier, Terrence Town

**Affiliations:** ^1^Regenerative Medicine Institute, Cedars-Sinai Medical CenterLos Angeles, CA, USA; ^2^Department of Biomedical Sciences, Cedars-Sinai Medical CenterLos Angeles, CA, USA; ^3^Department of Physiology and Biophysics, Zilkha Neurogenetic Institute, Keck School of Medicine of the University of Southern CaliforniaLos Angeles, CA, USA

**Keywords:** traumatic brain injury, Alzheimer disease, neuroinflammation, chronic traumatic encephalopathy, tauopathy, amyloid-beta peptides, neuronal loss, transgenic rat model

## Abstract

With as many as 300,000 United States troops in Iraq and Afghanistan having suffered head injuries (Miller, 2012), traumatic brain injury (TBI) has garnered much recent attention. While the cause and severity of these injuries is variable, severe cases can lead to lifelong disability or even death. While aging is the greatest risk factor for Alzheimer's disease (AD), it is now becoming clear that a history of TBI predisposes the individual to AD later in life (Sivanandam and Thakur, 2012). In this review article, we begin by defining hallmark pathological features of AD and the various forms of TBI. Putative mechanisms underlying the risk relationship between these two neurological disorders are then critically considered. Such mechanisms include precipitation and ‘spreading’ of cerebral amyloid pathology and the role of neuroinflammation. The combined problems of TBI and AD represent significant burdens to public health. A thorough, mechanistic understanding of the precise relationship between TBI and AD is of utmost importance in order to illuminate new therapeutic targets. Mechanistic investigations and the development of preclinical therapeutics are reliant upon a clearer understanding of these human diseases and accurate modeling of pathological hallmarks in animal systems.

## Introduction to traumatic brain injury

It is estimated that as many as 300,000 U.S. troops in Iraq and Afghanistan have suffered head injuries (Miller, 2012). In the general population, roughly 1.7 million brain injuries are reported, leading to more than 52,000 deaths (Faul et al., 2010). The cause and severity of these injuries is variable, but severe cases can lead to lifelong disability or even death. It is estimated that as many as 5.3 million people have traumatic brain injury (TBI)-associated disabilities. Moreover, TBI-associated direct and indirect costs are approximated to be more than 75 billion dollars a year (Coronado et al., 2012). Beyond the effects of acute injury, troubling new findings indicate that even minor brain injury can predispose to neurodegeneration and dementia in later life. While aging is generally accepted to be the greatest risk factor for Alzheimer's disease (AD), it is now widely recognized that a history of TBI is a key risk factor for the disease (Sivanandam and Thakur, 2012). For example, incidence of AD is significantly increased in individuals who have a documented history of TBI (Sivanandam and Thakur, 2012).

## Alzheimer's disease: severity of the problem

Largely due to population-wide increases in life-span, AD is rapidly becoming the public health crisis of our time. There are currently over three million Americans afflicted with the disease, a figure that is projected to increase to nearly nine million Americans and over 100 million world-wide by 2050 (Brookmeyer et al., 2007). Unfortunately, AD prevalence will continue to rise in parallel with the aging of the world's populations unless something is done (Brookmeyer et al., 2007). Because of the long prodromal phase leading to clinical manifestation of AD, TBI early in life would not impact AD diagnosis until decades later. At that point, the full impact of TBI-induced AD on soldiers and their families would place an unprecedented burden on the United States public health system.

AD is a devastating, mind-robbing neurodegenerative disease that is defined at autopsy by β-amyloid plaques [chiefly comprised of amyloid-β (Aβ) peptides], neurofibrillary tangles (NFTs), and widespread loss of cortical neurons (Selkoe, 2001). Although these features are pathognomonic of AD, Alois Alzheimer himself originally identified a third pathology—inflammation of the brain's glial supporting cells (Alzheimer et al., 1995). While one interpretation is that all forms of neuroinflammation are deleterious for the aging brain, we have hypothesized that re-balancing inflammatory signals as opposed to shutting them off completely might limit AD progression (Town et al., 2005; Weitz and Town, 2012). In that vein, it has been shown that genetic or pharmacologic blockade of a key pathway responsible for suppressing inflammation, the transforming growth factor-beta (TGF-β)-Smad 2/3 signaling cascade, reduces AD-like pathology. Specifically, peripheral blockade of the TGF-β-Smad 2/3 signaling pathway leads to brain penetration of peripheral macrophages and amelioration of the defining pathology of AD—β-amyloid plaques—in the Tg2576 transgenic mouse model of cerebral amyloidosis (Town et al., 2008; Rezai-Zadeh et al., 2009; Town, 2009; Gate et al., 2010). These results have importance, because there is currently no treatment or cure available for AD.

## Traumatic brain injury and Alzheimer's disease risk

The term “TBI” encompasses a wide variety of traumas. In fact, any form of brain injury is broadly classified as a TBI. Nevertheless, brain traumas can grossly be divided into two categories: (1) closed head injuries (where a rapid deceleration or blow to the head causes brain damage) or (2) penetrating head injuries (caused by a foreign object piercing the skull). Closed head injuries can come in the form of skull fractures, brain contusions caused by brain-skull impact, hematomas, and diffuse axonal injuries brought on by shearing forces. Notably, closed head injuries associated with concussions from contact sports and shockwave blasts from improvised explosive devices have garnered much recent attention. TBIs may range from mild to severe, with about 75% of injuries coming in the form of concussions or other mild TBIs (Hyder et al., 2007).

Pathological analyses of human TBI tissue have led to variable conclusions as compared with animal model studies. This is likely attributable to both the heterogeneity of the injury itself and methods of tissue collection. Nonetheless, several broad patterns of results have emerged. One of the most notable findings concerns the association between AD pathological features and TBI. For example, by examining cortical regions from TBI patients with survival times ranging from 4 h to several years, increased expression of the amyloid precursor protein (APP; which gives rise to the Aβ peptides that comprise senile plaques) has been demonstrated in the acute response to brain injury (Roberts et al., 1994; Graham et al., 1996). Another study reported that APP could be used as a general marker for axonal injury in human post-mortem material (Gentleman et al., 1993). More recently, Aβ deposits have been observed in roughly a third of TBI patients and as early as 2 h after injury (Ikonomovic et al., 2004). Overall, the conclusions were that Aβ plaques developed rapidly after injury, while NFTs formed during the chronic phase of disease (Ikonomovic et al., 2004). Follow-up studies have documented that severe TBI can induce Aβ42 (widely regarded as the more pathogenic species of the peptide), potentially leading to increased risk of AD later in life (DeKosky et al., 2007).

A very recent study examined survivors of a single TBI 1–47 years after the trauma, and reported that NFTs and Aβ pathology were present in approximately one-third of these patients. Such findings demonstrate the long-term consequences of a single TBI event (Johnson et al., 2012). On the other hand, more chronic, mild TBIs are associated with a distinct pathology, termed chronic traumatic encephalopathy (CTE) (McKee et al., 2010). Notably in CTE, NFTs are typically found with gliosis, but β-amyloid deposits are less obvious as compared with AD (Costanza et al., 2011). Unfortunately, CTE has become increasingly recognized in war veterans, boxers, and athletes in other impact sports (McKee et al., 2010; Costanza et al., 2011; Gavett et al., 2011; Stern et al., 2011; Goldstein et al., 2012; Miller, 2012; Shively et al., 2012; McKee et al., 2013). These troubling findings are no doubt cause for concern.

## Mechanisms to account for the risk relationship between traumatic brain injury and Alzheimer's disease

As mentioned above, TBI is a strong epigenetic risk factor for development of AD later in life. Strikingly, several defining AD pathological hallmarks have been observed following TBI in patient brains and in numerous TBI animal models. In addition to neuronal and synaptic loss (Kotapka et al., 1992; Smith et al., 1997; Maxwell et al., 2010), AD-characteristic lesions include accumulation of Aβ peptides, hyper-phosphorylated tau protein (the principle component of NFTs), and persistent microgliosis. A key question that arises from these observations is: what are the mechanism(s) responsible for development of AD in patients with a clinical history of TBI?

### Aβ pathology and spreading

Aβ deposits and widespread axonal Aβ accumulation have been found in patients' brains shortly after TBI (Roberts et al., 1991, 1994; Graham et al., 1995; Smith et al., 2003a; Ikonomovic et al., 2004; Uryu et al., 2007) and are still present many years after a single severe head trauma or repetitive mild TBIs (Tokuda et al., 1991; Johnson et al., 2012). Remarkably, following TBI, the major type of soluble and deposited Aβ peptide found in patients' brains is Aβ 42, well-known for its neurotoxicity and high propensity to aggregate (Gentleman et al., 1997; DeKosky et al., 2007). Several studies have described dramatic APP accumulation in swollen axons after TBI, which would provide an abundant source of substrate for Aβ production (Gentleman et al., 1993; Sherriff et al., 1994; Gorrie et al., 2002). Axonal swelling observed after TBI has been ascribed to cytoskeletal alteration and interruption of protein transport (Maxwell et al., 2003).

In an attempt to clarify mechanisms of plaque appearance after brain trauma, several non-transgenic rodent and rabbit models have been utilized. While these animal models have proved useful to characterize axonal Aβ accumulation after TBI, wild-type rodents and rabbits did not manifest cerebral β-amyloid plaques. This is likely owed to the fact that these TBI animal models have relatively low abundance of brain endogenous Aβ species that do not reach a critical threshold for aggregation (Lewen et al., 1995; Pierce et al., 1996; Bramlett et al., 1997; Hoshino et al., 1998; Iwata et al., 2002; Stone et al., 2002; Hamberger et al., 2003; Abrahamson et al., 2006). Another strategy has been to rely on transgenic mice that develop age-dependent Aβ plaque deposition. Unlike their wild-type counterparts, these animal models have contributed to our understanding of mechanisms of Aβ deposition after TBI. Like their non-transgenic counterparts, these transgenic mice manifest axonal Aβ post-TBI. However, and unlike wild-type animals, these transgenics demonstrate enhanced accumulation of β-amyloid plaques after TBI (Smith et al., 1998, 1999; Hartman et al., 2002; Uryu et al., 2002; Abrahamson et al., 2009). Moreover, studies in various animal models indicate that expression of amyloidogenic β- and γ-secretases and their substrate—APP—is increased after TBI, suggesting that Aβ peptides are generated *de novo* following brain trauma (Cribbs et al., 1996; Blasko et al., 2004; Chen et al., 2004; Nadler et al., 2008; Loane et al., 2009; Tran et al., 2011a; Yu et al., 2012). Thus, long-lasting elevation of Aβ following TBI is likely to result in Aβ pathology, up to and including senile plaque formation.

An important related concept is the idea of Aβ pathology “spreading.” Interestingly, studies from Mathias Jücker's laboratory and others have shown that intracerebral infusion of brain extracts containing aggregated Aβ can initiate Aβ deposition in brains of APP transgenic mice (Kane et al., 2000; Walker et al., 2002; Meyer-Luehmann et al., 2006; Eisele et al., 2009). Furthermore, it has been shown that Aβ seeds can migrate between axonally interconnected areas, suggesting that Aβ peptides can spread from the site of injection to other brain regions (Walker et al., 2002; Eisele et al., 2009). These results provide a potential mechanism for TBI-induced amyloid pathology spreading from the site of the TBI to other brain areas classically associated with AD-type pathological lesions but not directly subjected to the TBI.

TBI has also been shown to induce tauopathy. In that regard, it is important to note that a similar process has been described for spreading of NFTs by axonal transport after injection of abnormally folded tau filaments into a mouse model of cerebral amyloidosis (Clavaguera et al., 2009). Such findings suggest the possibility of abnormal tau protein seeds that spread following TBI. These results are summarized in Table 1.

**Table 1:** **Alzheimer's disease-type lesions induced by various TBIs in humans and in animal models**.

**AD-like lesions induced by TBI**	**Species**	**Type of injury involved**	**References**
Amyloidogenic APP processing and Aβ accumulation	Mouse	Focal	Cribbs et al., 1996
		CCI	Hartman et al., 2002; Abrahamson et al., 2006, 2009; Loane et al., 2009; Tran et al., 2011a; Yu et al., 2012
		Closed head	Nadler et al., 2008
	Rat	Mild compression contusion	Lewen et al., 1995
		Lateral fluid-percussion	Pierce et al., 1996; Bramlett et al., 1997; Hoshino et al., 1998; Iwata et al., 2002
		Cortical electro-coagulation	Luth et al., 2001
		Traumatic axonal	Stone et al., 2002
		CCI	Blasko et al., 2004
	Rabbit	Rotational acceleration	Hamberger et al., 2003
	Pig	Rotational acceleration	Smith et al., 1999; Chen et al., 2004
	Human	Single severe head	Roberts et al., 1991, 1994; Gentleman et al., 1993, 1997; Graham et al., 1995; Ikonomovic et al., 2004; DeKosky et al., 2007; Uryu et al., 2007; Johnson et al., 2012
		Dementia pugilistica	Tokuda et al., 1991; Schmidt et al., 2001
Tauopathy	Mouse	Repetitive mild	Yoshiyama et al., 2005; Ojo et al., 2013
		Blast and/or concussive	Goldstein et al., 2012
		CCI	Tran et al., 2011a
	Rat	Lateral fluid percussion	Hoshino et al., 1998
	Pig	Rotational acceleration	Smith et al., 1999
	Human	Repetitive mild trauma/Dementia pugilistica	Tokuda et al., 1991; McKenzie et al., 1996; Geddes et al., 1999
		Severe closed head	Zemlan et al., 1999
		Single acute brain	Smith et al., 2003b; Johnson et al., 2012
		Blast and/or concussive	Goldstein et al., 2012
Neuroinflammation	Mouse	Repetitive mild	Shitaka et al., 2011; Ojo et al., 2013
		Fluid percussion	Carbonell and Grady, 1999
		CCI	Israelsson et al., 2008
		Laser-induced focal ablation	Davalos et al., 2005
	Rat	CCI	Smith et al., 1997; Koshinaga et al., 2000
	Monkey	Surgical lesion	Nagamoto-Combs et al., 2007
	Human	Various	Gentleman et al., 2004; Morganti-Kossmann et al., 2007; Ramlackhansingh et al., 2011; Johnson et al., 2013
Neuronal loss/apoptosis	Mouse	CCI	Lewen et al., 2001; Yatsiv et al., 2005; Tehranian et al., 2006
		Weight-drop	Hutchison et al., 2001
	Rat	Fluid percussion injury	Cortez et al., 1989; Dietrich et al., 1994; Rink et al., 1995; Sinson et al., 1997; Yakovlev et al., 1997; Conti et al., 1998; Pierce et al., 1998; O'Dell et al., 2000; Raghupathi et al., 2002
		CCI	Sutton et al., 1993; Clark et al., 1997, 2001
		Weight-drop	Pravdenkova et al., 1996
	Human	Various	Mantyla, 1981; Bigler et al., 1992; Clark et al., 2000; Ng et al., 2000; Liou et al., 2003; Hausmann et al., 2004; Nathoo et al., 2004

AD, Alzheimer's disease; CCI, controlled cortical impact.

### Neuroinflammation

In patients' brains as well as in experimental animal models, TBI has been associated with microglial activation (Carbonell and Grady, 1999; Koshinaga et al., 2000; Davalos et al., 2005; Morganti-Kossmann et al., 2007; Ojo et al., 2013). The early phase of microglial activation in response to brain injury is accompanied by increased levels of interleukin-10 and TGF-β, which are generally regarded as anti-inflammatory cytokines that are capable of mediating neural protection and regeneration (Knoblach and Faden, 1998; Csuka et al., 1999; Tyor et al., 2002). Anti-inflammatory microglia with phagocytic properties have the potential to clear Aβ species and β-amyloid plaques; remarkably, Aβ-containing microglia have been found in association with plaques after TBI (Chen et al., 2009). Such findings suggest that microglia play a principle role in remodeling cerebral amyloid following brain injury (Giunta et al., 2008).

Depending on their activation state, microglia can be deleterious or beneficial in the context of cerebral amyloid deposition (Town et al., 2005; Weitz and Town, 2012). However, in rodents, primates and humans, microglial activation persists for months or even years after TBI, indicative of chronic neuroinflammation (Smith et al., 1997; Csuka et al., 2000; Gentleman et al., 2004; Nagamoto-Combs et al., 2007; Nagamoto-Combs and Combs, 2010; Ramlackhansingh et al., 2011; Shitaka et al., 2011). Chronic cerebral inflammation is typically associated with increased abundance of proinflammatory cytokines such as IL-1β, TNF-α and IL-6 and an array of chemokines (Stover et al., 2000; Morganti-Kossmann et al., 2001; Rothwell, 2003; Dietrich et al., 2004; Israelsson et al., 2008). This phenotype is remarkably similar to the low-level pro-inflammatory, chronic microglial activation state that occurs in AD and ultimately fails to restrict amyloid deposition. Additionally, it has been extensively reported that aging microglia undergo structural deterioration and cellular senescence, which likely predicts poor Aβ clearance aptitude (Flanary and Streit, 2004; Fiala et al., 2005; Hickman et al., 2008; Njie et al., 2012). Furthermore, TBI is classically followed by oxidative stress and hypoxia, which are known to stimulate microglia and astrocytes and induce release of IL-1β, TNF-α, interferon-γ and IL-6 (Luth et al., 2001). These pro-inflammatory cytokines can stimulate γ-secretase activity and enhance APP levels and amyloidogenic APP processing, potentially exacerbating Aβ pathology (Tamagno et al., 2003; Blasko et al., 2004; Liao et al., 2004; Rogers et al., 2008; Agostinho et al., 2010). In addition, increased expression of presenilin-1 and nicastrin in TBI-activated microglia has been described in mice, reinforcing the probable implication of microglia in post-injury Aβ pathology (Liao et al., 2004; Nadler et al., 2008). Altogether, these mechanisms could perpetrate a chronic vicious cycle involving inefficient activation of microglia, cerebral Aβ accumulation and spreading, and development of AD-type pathology. In summary then, the early inflammatory response after TBI may negatively impact AD pathology later on.

## Animal models: present and future

The development of clinically-relevant animal models is critically important to enable future study at the intersection of TBI and AD research. Animal models of AD fail to exhibit some of the key pathological earmarks of the human syndrome, even after significant brain injury (Uryu et al., 2002; Tran et al., 2011a,b). For example, one of the principle symptoms lacking in transgenic mouse models constructed with mutations that cause early-onset familial AD is fulminant neuronal loss (Duyckaerts et al., 2008). For while most transgenic mouse models display amyloid deposition, and some exhibit tau pathology, almost all do not have appreciable neuronal death (Duyckaerts et al., 2008). For instance, the principle readouts after TBI in a widely-used mouse model of AD, the 3× Tg-AD mouse, consist primarily of hyperphosphorylated tau and β-amyloid plaques, because the model does not allow insight into the widespread cortical neuronal loss observed in the human disease (Tran et al., 2011a).

**Figure 1:**
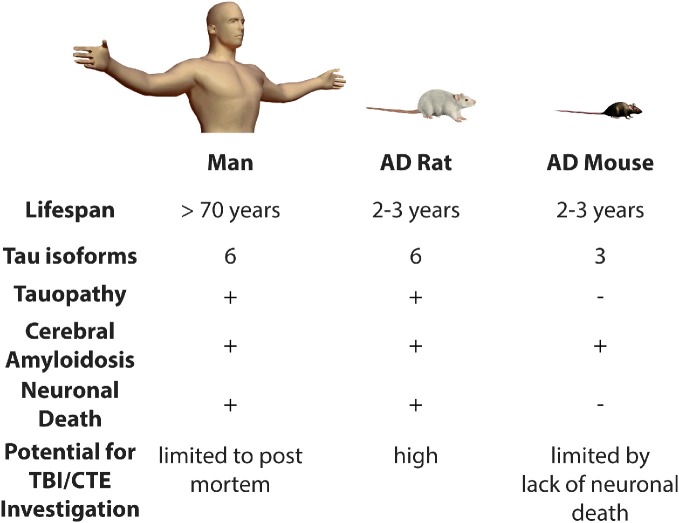
**Modeling the risk relationship between traumatic brain injury and Alzheimer's disease.** Presence (+) or absence (−) of various pathological features is indicated. AD, Alzheimer's disease; TBI, Traumatic Brain Injury; CTE, Chronic Traumatic Encephalopathy.

By contrast, we have recently published a novel rat transgenic model of AD, line TgF344-AD. This transgenic line expresses mutant human APP and presenilin-1, which are each independent genetic causes of early-onset familial AD. Notably, this rat displays the full spectrum of human AD hallmarks, including cerebral amyloidosis, tauopathy, gliosis, and most importantly, large-scale apoptotic loss of neurons in cortical and hippocampal regions. Moreover, these animals display significant age-dependent cognitive disturbance (Cohen et al., 2013). The precise reason(s) for the differences between this new transgenic rat model and analogous mouse models are unclear. Rats are four-to-five million years closer to humans on the evolutionary tree than mice. In addition, rats, like humans and unlike mice, have all six tau isoforms. Therefore, rats have a physiology that is more similar to the human and may be more permissive to neurodegenerative disease. For these reasons, it will be highly informative to test whether TBI precipitates earlier neuronal loss and tauopathy in this line of rats. Moreover, the behavioral correlates of neuronal damage and loss can be carefully related in a way that more closely approximates human trauma and associated cognitive decline. Specifically, if TBI leads to neuronal loss in the long term, these rats might be used to determine if therapeutic intervention(s) could be introduced to attenuate or prevent neurodegeneration and cognitive impairment (Figure 1).

## Conclusions and future directions

The correlation between brain injury and neurodegenerative disease is now well-established (Szczygielski et al., 2005; Shively et al., 2012). The combined problems of TBI and AD will become increasingly significant burdens to society. Both diseases will require early identification in the form of imaging or biomarkers to allow for therapeutic intervention at the earliest possible stages. Unfortunately, a treatment or therapy does not currently exist for either disease. A thorough, mechanistic understanding of the precise relationship between TBI and AD is of utmost importance in order to illuminate new therapeutic targets. However, as we have highlighted, key questions remain regarding the precise mechanisms linking the many forms of brain injury with precipitation of AD-type neurodegeneration.

These mechanistic investigations and the development of pre-clinical therapeutics will rely critically on a clearer understanding of both human pathologies. A key limiting factor is the large gap in our knowledge of the link between post-mortem observations in humans after TBI with animal model systems. Part of the uncertainty can be attributed to limitations inherent to experimental models of TBI. Therefore, it is expected that more precise modeling of pathological hallmarks in animal models will allow us to fill the knowledge gap. Specifically, it will be critical to develop models that accurately mimic the forces impacting the human brain under a variety of circumstances (Morales et al., 2005; Blennow et al., 2012).

### Conflict of interest statement

The authors declare that the research was conducted in the absence of any commercial or financial relationships that could be construed as a potential conflict of interest.

## References

[B1] Abrahamson E. E. Ikonomovic M. D. Ciallella J. R. Hope C. E. Paljug W. R. Isanski B. A. (2006). Caspase inhibition therapy abolishes brain trauma-induced increases in *Abeta peptide*: implications for clinical outcome. Exp. Neurol. 197, 437–450 10.1016/j.expneurol.2005.10.011 16300758

[B2] Abrahamson E. E. Ikonomovic M. D. Dixon C. E. DeKosky S. T. (2009). Simvastatin therapy prevents brain trauma-induced increases in beta-amyloid peptide levels. Ann. Neurol. 66, 407–414 10.1002/ana.21731 19798641

[B3] Agostinho P., R. Cunha A. Oliveira C. (2010). Neuroinflammation, oxidative stress and the pathogenesis of Alzheimer's disease. Curr. Pharm. Des. 16, 2766–2778 10.2174/138161210793176572 20698820

[B4] Alzheimer A. Stelzmann R. A. Schnitzlein H. N. Murtagh F. R. (1995). An English translation of Alzheimer's 1907 paper, “Uber eine eigenartige Erkankung der Hirnrinde.” Clin. Anat. 8, 429–431 10.1002/ca.980080612 8713166

[B5] Bigler E. D. Kurth S. M. Blatter D. Abildskov T. J. (1992). Degenerative changes in traumatic brain injury: post-injury magnetic resonance identified ventricular expansion compared to pre-injury levels. Brain Res. Bull. 28, 651–653 10.1016/0361-9230(92)90119-I 1617451

[B6] Blasko I. Beer R. Bigl M. Apelt J. Franz G. Rudzki D. (2004). Experimental traumatic brain injury in rats stimulates the expression, production and activity of Alzheimer's disease beta-secretase (BACE-1). J. Neural Transm. 111, 523–536 10.1007/s00702-003-0095-6 15057522

[B7] Blasko I. Stampfer-Kountchev M. Robatscher P. Veerhuis R. Eikelenboom P. Grubeck-Loebenstein B. (2004). How chronic inflammation can affect the brain and support the development of Alzheimer's disease in old age: the role of microglia and astrocytes. Aging Cell 3, 169–176 10.1111/j.1474-9728.2004.00101.x 15268750

[B8] Blennow K. Hardy J. Zetterberg H. (2012). The neuropathology and neurobiology of traumatic brain injury. Neuron 76, 886–899 10.1016/j.neuron.2012.11.021 23217738

[B9] Bramlett H. M. Kraydieh S. Green E. J. Dietrich W. D. (1997). Temporal and regional patterns of axonal damage following traumatic brain injury: a beta-amyloid precursor protein immunocytochemical study in rats. J. Neuropathol. Exp. Neurol. 56, 1132–1141 10.1097/00005072-199710000-00007 9329457

[B10] Brookmeyer R. Johnson E. Ziegler-Graham K. Arrighi H. M. (2007). Forecasting the global burden of Alzheimer's disease. Alzheimers Dement 3, 186–191 10.1016/j.jalz.2007.04.381 19595937

[B11] Carbonell W. S. Grady M. S. (1999). Regional and temporal characterization of neuronal, glial, and axonal response after traumatic brain injury in the mouse. Acta Neuropathol. 98, 396–406 10.1007/s004010051100 10502046

[B12] Chen X. H. Johnson V. E. Uryu K. Trojanowski J. Q. Smith D. H. (2009). A lack of amyloid beta plaques despite persistent accumulation of amyloid beta in axons of long-term survivors of traumatic brain injury. Brain Pathol. 19, 214–223 10.1111/j.1750-3639.2008.00176.x 18492093 PMC3014260

[B13] Chen X. H. Siman R. Iwata A. Meaney D. F. Trojanowski J. Q. Smith D. H. (2004). Long-term accumulation of amyloid-beta, beta-secretase, presenilin-1, and caspase-3 in damaged axons following brain trauma. Am. J. Pathol. 165, 357–371 10.1016/S0002-9440(10)63303-2 15277212 PMC1618579

[B14] Clark R. S. Kochanek P. M. Adelson P. D. Bell M. J. Carcillo J. A. Chen M. (2000). Increases in bcl-2 protein in cerebrospinal fluid and evidence for programmed cell death in infants and children after severe traumatic brain injury. J. Pediatr. 137, 197–204 10.1067/mpd.2000.106903 10931412

[B16] Clark R. S. Kochanek P. M. Dixon C. E. Chen M. Marion D. W. Heineman S. (1997). Early neuropathologic effects of mild or moderate hypoxemia after controlled cortical impact injury in rats. J. Neurotrauma 14, 179–189 10.1089/neu.1997.14.179 9151767

[B15] Clark R. S. B. Chen M. Kochanek P. M. Watkins S. C. Jin K. L. Draviam R. (2001). Detection of single- and double-strand DNA breaks after traumatic brain injury in rats: comparison of *in situ* labeling techniques using DNA polymerase I, the Klenow fragment of DNA polymerase I, and terminal deoxynucleotidyl transferase. J. Neurotrauma 18, 675–689 10.1089/089771501750357627 11497094

[B17] Clavaguera F. Bolmont T. Crowther R. A. Abramowski D. Frank S. Probst A. (2009). Transmission and spreading of tauopathy in transgenic mouse brain. Nat. Cell Biol. 11, 909–913 10.1038/ncb1901 19503072 PMC2726961

[B18] Cohen R. M. Rezai-Zadeh K. Weitz T. M. Rentsendorj A. Gate D. Spivak I. (2013). A transgenic Alzheimer rat with plaques, tau pathology, behavioral impairment, oligomeric abeta, and Frank neuronal loss. J. Neurosci. 33, 6245–6256 10.1523/JNEUROSCI.3672-12.2013 23575824 PMC3720142

[B19] Conti A. C. Raghupathi R. Trojanowski J. Q. McIntosh T. K. (1998). Experimental brain injury induces regionally distinct apoptosis during the acute and delayed post-traumatic period. J. Neurosci. 18, 5663–5672 9671657 10.1523/JNEUROSCI.18-15-05663.1998 PMC6793063

[B20] Coronado V. G. McGuire L. C. Faul M. Sugerman D. E. Pearson W. S. (2012). “Traumatic brain injury epidemiology and public health issues,” in Brain Injury Medicine: Principles and Practice, 84

[B21] Cortez S. C. McIntosh T. K. Noble L. J. (1989). Experimental fluid percussion brain injury: vascular disruption and neuronal and glial alterations. Brain Res. 482, 271–282 10.1016/0006-8993(89)91190-6 2706487

[B22] Costanza A. Weber K. Gandy S. Bouras C. Hof P. R. Giannakopoulos P. (2011). Review: Contact sport-related chronic traumatic encephalopathy in the elderly: clinical expression and structural substrates. Neuropathol. Appl. Neurobiol. 37, 570–584 10.1111/j.1365-2990.2011.01186.x 21696410 PMC3166385

[B23] Cribbs D. H. Chen L. S. Cotman C. W. LaFerla F. M. (1996). Injury induces presenilin-1 gene expression in mouse brain. Neuroreport 7, 1773–1776 10.1097/00001756-199607290-00016 8905662

[B24] Csuka E. Hans V. H. Ammann E. Trentz O. Kossmann T. Morganti-Kossmann M. C. (2000). Cell activation and inflammatory response following traumatic axonal injury in the rat. Neuroreport 11, 2587–2590 10.1097/00001756-200008030-00047 10943727

[B25] Csuka E. Morganti-Kossmann M. C. Lenzlinger P. M. Joller H. Trentz O. Kossmann T. (1999). IL-10 levels in cerebrospinal fluid and serum of patients with severe traumatic brain injury: relationship to IL-6, TNF-alpha, TGF-beta1 and blood-brain barrier function. J. Neuroimmunol. 101, 211–221 10.1016/S0165-5728(99)00148-4 10580806

[B26] Davalos D. Grutzendler J. Yang G. Kim J. V. Zuo Y. Jung S. (2005). ATP mediates rapid microglial response to local brain injury *in vivo*. Nat. Neurosci. 8, 752–758 10.1038/nn1472 15895084

[B27] DeKosky S. T. Abrahamson E. E. Ciallella J. R. Paljug W. R. Wisniewski S. R. Clark R. S. (2007). Association of increased cortical soluble abeta42 levels with diffuse plaques after severe brain injury in humans. Arch. Neurol. 64, 541–544 10.1001/archneur.64.4.541 17420316

[B28] Dietrich W. D. Alonso O. Busto R. Globus M. Y. Ginsberg M. D. (1994). Post-traumatic brain hypothermia reduces histopathological damage following concussive brain injury in the rat. Acta Neuropathol. 87, 250–258 10.1007/BF00296740 8009957

[B29] Dietrich W. D. Chatzipanteli K. Vitarbo E. Wada K. Kinoshita K. (2004). The role of inflammatory processes in the pathophysiology and treatment of brain and spinal cord trauma. Acta Neurochir. Suppl. 89, 69–74 10.1007/978-3-7091-0603-7_9 15335103

[B30] Duyckaerts C. Potier M. C. Delatour B. (2008). Alzheimer disease models and human neuropathology: similarities and differences. Acta Neuropathol. 115, 5–38 10.1007/s00401-007-0312-8 18038275 PMC2100431

[B31] Eisele Y. S. Bolmont T. Heikenwalder M. Langer F. Jacobson L. H. Yan Z. X. (2009). Induction of cerebral beta-amyloidosis: intracerebral versus systemic Abeta inoculation. Proc. Natl. Acad. Sci. U.S.A. 106, 12926–12931 10.1073/pnas.0903200106 19622727 PMC2722323

[B32] Faul M. Xu L. Wald M. M. Coronado V. G. (2010). Traumatic Brain Injury in the United States: Emergency Department Visits, Hospitalizations, and Deaths. Centers for Disease Control and Prevention, National Center for Injury Prevention and Control. Atlanta (GA).

[B33] Fiala M. Lin J. Ringman J. Kermani-Arab V. Tsao G. Patel A. (2005). Ineffective phagocytosis of amyloid-beta by macrophages of Alzheimer's disease patients. J. Alzheimers Dis. 7, 221–232 discussion: 255–262. 16006665 10.3233/jad-2005-7304

[B34] Flanary B. E. Streit W. J. (2004). Progressive telomere shortening occurs in cultured rat microglia, but not astrocytes. Glia 45, 75–88 10.1002/glia.10301 14648548

[B35] Gate D. Rezai-Zadeh K. Jodry D. Rentsendorj A. Town T. (2010). Macrophages in Alzheimer's disease: the blood-borne identity. J. Neural Transm. 117, 961–970 10.1007/s00702-010-0422-7 20517700 PMC2917548

[B36] Gavett B. E. Stern R. A. McKee A. C. (2011). Chronic traumatic encephalopathy: a potential late effect of sport-related concussive and subconcussive head trauma. Clin. Sports Med. 30, 179–188 xi. 10.1016/j.csm.2010.09.007 21074091 PMC2995699

[B37] Geddes J. F. Vowles G. H. Nicoll J. A. Revesz T. (1999). Neuronal cytoskeletal changes are an early consequence of repetitive head injury. Acta Neuropathol. 98, 171–178 10.1007/s004010051066 10442557

[B38] Gentleman S. M. Greenberg B. D. Savage M. J. Noori M. Newman S. J. Roberts G. W. (1997). A beta 42 is the predominant form of amyloid beta-protein in the brains of short-term survivors of head injury. Neuroreport 8, 1519–1522 10.1097/00001756-199704140-00039 9172166

[B39] Gentleman S. M. Leclercq P. D. Moyes L. Graham D. I. Smith C. Griffin W. S. (2004). Long-term intracerebral inflammatory response after traumatic brain injury. Forensic Sci. Int. 146, 97–104 10.1016/j.forsciint.2004.06.027 15542269

[B40] Gentleman S. M. Nash M. J. Sweeting C. J. Graham D. I. Roberts G. W. (1993). Beta-amyloid precursor protein (beta APP) as a marker for axonal injury after head injury. Neurosci. Lett. 160, 139–144 10.1016/0304-3940(93)90398-5 8247344

[B41] Giunta B. Fernandez F. Nikolic W. V. Obregon D. Rrapo E. Town T. (2008). Inflammaging as a prodrome to Alzheimer's disease. J. Neuroinflamm. 5:51 10.1186/1742-2094-5-51 19014446 PMC2615427

[B42] Goldstein L. E. Fisher A. M. Tagge C. A. Zhang X. L. Velisek L. Sullivan J. A. (2012). Chronic traumatic encephalopathy in blast-exposed military veterans and a blast neurotrauma mouse model. Sci. Transl. Med. 4:134ra160 10.1126/scitranslmed.3003716 PMC3739428 22593173

[B43] Gorrie C. Oakes S. Duflou J. Blumbergs P. Waite P. M. (2002). Axonal injury in children after motor vehicle crashes: extent, distribution, and size of axonal swellings using beta-APP immunohistochemistry. J. Neurotrauma 19, 1171–1182 10.1089/08977150260337976 12427326

[B44] Graham D. I. Gentleman S. M. Lynch A. Roberts G. W. (1995). Distribution of beta-amyloid protein in the brain following severe head injury. Neuropathol. Appl. Neurobiol. 21, 27–34 10.1111/j.1365-2990.1995.tb01025.x 7770117

[B45] Graham D. I. Gentleman S. M. Nicoll J. A. Royston M. C. McKenzie J. E. Roberts G. W. (1996). Altered beta-APP metabolism after head injury and its relationship to the aetiology of Alzheimer's disease. Acta Neurochir. Suppl. 66, 96–102 8780805 10.1007/978-3-7091-9465-2_17

[B46] Hamberger A. Huang Y. L. Zhu H. Bao F. Ding M. Blennow K. (2003). Redistribution of neurofilaments and accumulation of beta-amyloid protein after brain injury by rotational acceleration of the head. J. Neurotrauma 20, 169–178 10.1089/08977150360547080 12675970

[B47] Hartman R. E. Laurer H. Longhi L. Bales K. R. Paul S. M. McIntosh T. K. (2002). Apolipoprotein E4 influences amyloid deposition but not cell loss after traumatic brain injury in a mouse model of Alzheimer's disease. J. Neurosci. 22, 10083–10087 12451108 10.1523/JNEUROSCI.22-23-10083.2002 PMC6758744

[B48] Hausmann R. Biermann T. Wiest I. Tubel J. Betz P. (2004). Neuronal apoptosis following human brain injury. Int. J. Legal Med. 118, 32–36 10.1007/s00414-003-0413-4 14625778

[B49] Hickman S. E. Allison E. K. El Khoury J. (2008). Microglial dysfunction and defective beta-amyloid clearance pathways in aging Alzheimer's disease mice. J. Neurosci. 28, 8354–8360 10.1523/JNEUROSCI.0616-08.2008 18701698 PMC2597474

[B50] Hoshino S. Tamaoka A. Takahashi M. Kobayashi S. Furukawa T. Oaki Y. (1998). Emergence of immunoreactivities for phosphorylated tau and amyloid-beta protein in chronic stage of fluid percussion injury in rat brain. Neuroreport 9, 1879–1883 10.1097/00001756-199806010-00039 9665619

[B51] Hutchison J. S. Derrane R. E. Johnston D. L. Gendron N. Barnes D. Fliss H. (2001). Neuronal apoptosis inhibitory protein expression after traumatic brain injury in the mouse. J. Neurotrauma 18, 1333–1347 10.1089/08977150152725632 11780864

[B52] Hyder A. A. Wunderlich C. A. Puvanachandra P. Gururaj G. Kobusingye O. C. (2007). The impact of traumatic brain injuries: a global perspective. Neuro Rehabil. 22, 341–353 18162698

[B53] Ikonomovic M. D. Uryu K. Abrahamson E. E. Ciallella J. R. Trojanowski J. Q. Lee V. M. (2004). Alzheimer's pathology in human temporal cortex surgically excised after severe brain injury. Exp. Neurol. 190, 192–203 10.1016/j.expneurol.2004.06.011 15473992

[B54] Israelsson C. Bengtsson H. Kylberg A. Kullander K. Lewen A. Hillered L. (2008). Distinct cellular patterns of upregulated chemokine expression supporting a prominent inflammatory role in traumatic brain injury. J. Neurotrauma 25, 959–974 10.1089/neu.2008.0562 18665806

[B55] Iwata A. Chen X. H. McIntosh T. K. Browne K. D. Smith D. H. (2002). Long-term accumulation of amyloid-beta in axons following brain trauma without persistent upregulation of amyloid precursor protein genes. J. Neuropathol. Exp. Neurol. 61, 1056–1068 12484568 10.1093/jnen/61.12.1056

[B56] Johnson V. E. Stewart J. E. Begbie F. D. Trojanowski J. Q. Smith D. H. Stewart W. (2013). Inflammation and white matter degeneration persist for years after a single traumatic brain injury. Brain 136(Pt 1), 28–42 23365092 10.1093/brain/aws322 PMC3562078

[B57] Johnson V. E. Stewart W. Smith D. H. (2012). Widespread tau and amyloid-beta pathology many years after a single traumatic brain injury in humans. Brain Pathol. 22, 142–149 10.1111/j.1750-3639.2011.00513.x 21714827 PMC3979351

[B58] Kane M. D. Lipinski W. J. Callahan M. J. Bian F. Durham R. A. Schwarz R. D. (2000). Evidence for seeding of beta-amyloid by intracerebral infusion of Alzheimer brain extracts in beta-amyloid precursor protein-transgenic mice. J. Neurosci. 20, 3606–3611 10804202 10.1523/JNEUROSCI.20-10-03606.2000 PMC6772682

[B59] Knoblach S. M. Faden A. I. (1998). Interleukin-10 improves outcome and alters proinflammatory cytokine expression after experimental traumatic brain injury. Exp. Neurol. 153, 143–151 10.1006/exnr.1998.6877 9743576

[B60] Koshinaga M. Katayama Y. Fukushima M. Oshima H. Suma T. Takahata T. (2000). Rapid and widespread microglial activation induced by traumatic brain injury in rat brain slices. J. Neurotrauma 17, 185–192 10.1089/neu.2000.17.185 10757324

[B61] Kotapka M. J. Graham D. I. Adams J. H. Gennarelli T. A. (1992). Hippocampal pathology in fatal non-missile human head injury. Acta Neuropathol. 83, 530–534 10.1007/BF00310031 1621508

[B62] Lewen A. Li G. L. Nilsson P. Olsson Y. Hillered L. (1995). Traumatic brain injury in rat produces changes of beta-amyloid precursor protein immunoreactivity. Neuroreport 6, 357–360 10.1097/00001756-199501000-00032 7756628

[B63] Lewen A. Sugawara T. Gasche Y. Fujimura M. Chan P. H. (2001). Oxidative cellular damage and the reduction of APE/Ref-1 expression after experimental traumatic brain injury. Neurobiol. Dis. 8, 380–390 10.1006/nbdi.2001.0396 11447995

[B64] Liao Y. F. Wang B. J. Cheng H. T. Kuo L. H. Wolfe M. S. (2004). Tumor necrosis factor-alpha, interleukin-1beta, and interferon-gamma stimulate gamma-secretase-mediated cleavage of amyloid precursor protein through a JNK-dependent MAPK pathway. J. Biol. Chem. 279, 49523–49532 10.1074/jbc.M402034200 15347683

[B65] Liou A. K. Clark R. S. Henshall D. C. Yin X. M. Chen J. (2003). To die or not to die for neurons in ischemia, traumatic brain injury and epilepsy: a review on the stress-activated signaling pathways and apoptotic pathways. Prog. Neurobiol. 69, 103–142 10.1016/S0301-0082(03)00005-4 12684068

[B66] Loane D. J. Pocivavsek A. Moussa C. E. Thompson R. Matsuoka Y. Faden A. I. (2009). Amyloid precursor protein secretases as therapeutic targets for traumatic brain injury. Nat. Med. 15, 377–379 10.1038/nm.1940 19287391 PMC2844765

[B67] Luth H. J. Holzer M. Gartner U. Staufenbiel M. Arendt T. (2001). Expression of endothelial and inducible NOS-isoforms is increased in Alzheimer's disease, in APP23 transgenic mice and after experimental brain lesion in rat: evidence for an induction by amyloid pathology. Brain Res. 913, 57–67 10.1016/S0006-8993(01)02758-5 11532247

[B68] Mantyla M. (1981). Post-traumatic cerebral atrophy. A study on brain-injured veterans on the Finnish wars of 1939-40 and 1941-45. Ann. Clin. Res. 13, 1–47 7283378

[B69] Maxwell W. L. Domleo A. McColl G. Jafari S. S. Graham D. I. (2003). Post-acute alterations in the axonal cytoskeleton after traumatic axonal injury. J. Neurotrauma 20, 151–168 10.1089/08977150360547071 12675969

[B70] Maxwell W. L. MacKinnon M. A. Stewart J. E. Graham D. I. (2010). Stereology of cerebral cortex after traumatic brain injury matched to the Glasgow outcome score. Brain 133(Pt 1), 139–160 10.1093/brain/awp264 19897544

[B71] McKee A. C. Gavett B. E. Stern R. A. Nowinski C. J. Cantu R. C. Kowall N. W. (2010). TDP-43 proteinopathy and motor neuron disease in chronic traumatic encephalopathy. J. Neuropathol. Exp. Neurol. 69, 918–929 10.1097/NEN.0b013e3181ee7d85 20720505 PMC2951281

[B72] McKee A. C. Stein T. D. Nowinski C. J. Stern R. A. Daneshvar D. H. Alvarez V. E. (2013). The spectrum of disease in chronic traumatic encephalopathy. Brain 136(Pt 1), 43–64 23208308 10.1093/brain/aws307 PMC3624697

[B73] McKenzie J. E. Roberts G. W. Royston M. C. (1996). Comparative investigation of neurofibrillary damage in the temporal lobe in Alzheimer's disease, Down's syndrome and dementia pugilistica. Neurodegeneration 5, 259–264 10.1006/neur.1996.0034 8910904

[B74] Meyer-Luehmann M. Coomaraswamy J. Bolmont T. Kaeser S. Schaefer C. Kilger E. (2006). Exogenous induction of cerebral beta-amyloidogenesis is governed by agent and host. Science 313, 1781–1784 10.1126/science.1131864 16990547

[B75] Miller G. (2012). Neuropathology. Blast injuries linked to neurodegeneration in veterans. Science 336, 790–791 10.1126/science.336.6083.790 22605724

[B76] Morales D. M. Marklund N. Lebold D. Thompson H. J. Pitkanen A. Maxwell W. L. (2005). Experimental models of traumatic brain injury: do we really need to build a better mousetrap? Neuroscience 136, 971–989 10.1016/j.neuroscience.2005.08.030 16242846

[B77] Morganti-Kossmann M. C. Rancan M. Otto V. I. Stahel P. F. Kossmann T. (2001). Role of cerebral inflammation after traumatic brain injury: a revisited concept. Shock 16, 165–177 10.1097/00024382-200116030-00001 11531017

[B78] Morganti-Kossmann M. C. Satgunaseelan L. Bye N. Kossmann T. (2007). Modulation of immune response by head injury. Injury 38, 1392–1400 10.1016/j.injury.2007.10.005 18048036

[B79] Nadler Y. Alexandrovich A. Grigoriadis N. Hartmann T. Rao K. S. Shohami E. (2008). Increased expression of the gamma-secretase components presenilin-1 and nicastrin in activated astrocytes and microglia following traumatic brain injury. Glia 56, 552–567 10.1002/glia.20638 18240300

[B80] Nagamoto-Combs K. Combs C. K. (2010). Microglial phenotype is regulated by activity of the transcription factor, NFAT (nuclear factor of activated T cells). J. Neurosci. 30, 9641–9646 20631193 10.1523/JNEUROSCI.0828-10.2010 PMC2914496

[B81] Nagamoto-Combs K. McNeal D. W. Morecraft R. J. Combs C. K. (2007). Prolonged microgliosis in the rhesus monkey central nervous system after traumatic brain injury. J. Neurotrauma 24, 1719–1742 10.1089/neu.2007.0377 18001202

[B82] Nathoo N. Narotam P. K. Agrawal D. K. Connolly C. A. van Dellen J. R. Barnett G. H. (2004). Influence of apoptosis on neurological outcome following traumatic cerebral contusion. J. Neurosurg. 101, 233–240 10.3171/jns.2004.101.2.0233 15309913

[B83] Ng I. Yeo T. T. Tang W. Y. Soong R. Ng P. Y. Smith D. R. (2000). Apoptosis occurs after cerebral contusions in humans. Neurosurgery 46, 949–956 10764270 10.1097/00006123-200004000-00034

[B84] Njie E. G. Boelen E. Stassen F. R. Steinbusch H. W. Borchelt D. R. Streit W. J. (2012). Ex vivo cultures of microglia from young and aged rodent brain reveal age-related changes in microglial function. Neurobiol. Aging 33, 195, e191–e112. 20580465 10.1016/j.neurobiolaging.2010.05.008 PMC4162517

[B85] O'Dell D. M. Muir J. K. Zhang C. Bareyre F. M. Saatman K. E. Raghupathi R. (2000). Lubeluzole treatment does not attenuate neurobehavioral dysfunction or CA3 hippocampal neuronal loss following traumatic brain injury in rats. Restor. Neurol. Neurosci. 16, 127–134 12671215

[B86] Ojo J. O. Mouzon B. Greenberg M. B. Bachmeier C. Mullan M. Crawford F. (2013). Repetitive mild traumatic brain injury augments tau pathology and glial activation in aged hTau mice. J. Neuropathol. Exp. Neurol. 72, 137–151 10.1097/NEN.0b013e3182814cdf 23334597

[B87] Pierce J. E. Smith D. H. Trojanowski J. Q. McIntosh T. K. (1998). Enduring cognitive, neurobehavioral and histopathological changes persist for up to one year following severe experimental brain injury in rats. Neuroscience 87, 359–369 10.1016/S0306-4522(98)00142-0 9740398

[B88] Pierce J. E. Trojanowski J. Q. Graham D. I. Smith D. H. McIntosh T. K. (1996). Immunohistochemical characterization of alterations in the distribution of amyloid precursor proteins and beta-amyloid peptide after experimental brain injury in the rat. J. Neurosci. 16, 1083–1090 8558237 10.1523/JNEUROSCI.16-03-01083.1996 PMC6578806

[B89] Pravdenkova S. V. Basnakian A. G. James S. J. Andersen B. J. (1996). DNA fragmentation and nuclear endonuclease activity in rat brain after severe closed head injury. Brain Res. 729, 151–155 10.1016/0006-8993(96)00222-3 8876983

[B90] Raghupathi R. Conti A. C. Graham D. I. Krajewski S. Reed J. C. Grady M. S. (2002). Mild traumatic brain injury induces apoptotic cell death in the cortex that is preceded by decreases in cellular Bcl-2 immunoreactivity. Neuroscience 110, 605–616 10.1016/S0306-4522(01)00461-4 11934469

[B91] Ramlackhansingh A. F. Brooks D. J. Greenwood R. J. Bose S. K. Turkheimer F. E. Kinnunen K. M. (2011). Inflammation after trauma: microglial activation and traumatic brain injury. Ann. Neurol. 70, 374–383 10.1002/ana.22455 21710619

[B92] Rezai-Zadeh K. Gate D. Town T. (2009). CNS infiltration of peripheral immune cells: D-Day for neurodegenerative disease? J. Neuroimmune Pharmacol. 4, 462–475 10.1007/s11481-009-9166-2 19669892 PMC2773117

[B93] Rink A. Fung K. M. Trojanowski J. Q. Lee V. M. Neugebauer E. McIntosh T. K. (1995). Evidence of apoptotic cell death after experimental traumatic brain injury in the rat. Am. J. Pathol. 147, 1575–1583 7495282 PMC1869937

[B94] Roberts G. W. Gentleman S. M. Lynch A. Graham D. I. (1991). beta A4 amyloid protein deposition in brain after head trauma. Lancet 338, 1422–1423 10.1016/0140-6736(91)92724-G 1683421

[B95] Roberts G. W. Gentleman S. M. Lynch A. Murray L. Landon M. Graham D. I. (1994). Beta amyloid protein deposition in the brain after severe head injury: implications for the pathogenesis of Alzheimer's disease. J. Neurol. Neurosurg. Psychiatry 57, 419–425 10.1136/jnnp.57.4.419 8163989 PMC1072869

[B96] Rogers J. T. Bush A. I. Cho H. H. Smith D. H. Thomson A. M. Friedlich A. L. (2008). Iron and the translation of the amyloid precursor protein (APP) and ferritin mRNAs: riboregulation against neural oxidative damage in Alzheimer's disease. Biochem. Soc. Trans. 36(Pt 6), 1282–1287 10.1042/BST0361282 19021541 PMC2746665

[B97] Rothwell N. (2003). Interleukin-1 and neuronal injury: mechanisms, modification, and therapeutic potential. Brain Behav. Immun. 17, 152–157 10.1016/S0889-1591(02)00098-3 12706413

[B98] Schmidt M. L. Zhukareva V. Newell K. L. Lee V. M. Trojanowski J. Q. (2001). Tau isoform profile and phosphorylation state in dementia pugilistica recapitulate Alzheimer's disease. Acta Neuropathol. 101, 518–524 11484824 10.1007/s004010000330

[B99] Selkoe D. J. (2001). Alzheimer's disease: genes, proteins, and therapy. Physiol. Rev. 81, 741–766 11274343 10.1152/physrev.2001.81.2.741

[B100] Sherriff F. E., L. R. Bridges Sivaloganathan S. (1994). Early detection of axonal injury after human head trauma using immunocytochemistry for beta-amyloid precursor protein. Acta Neuropathol. 87, 55–62 10.1007/BF00386254 8140894

[B101] Shitaka Y. Tran H. T. Bennett R. E. Sanchez L. Levy M. A. Dikranian K. (2011). Repetitive closed-skull traumatic brain injury in mice causes persistent multifocal axonal injury and microglial reactivity. J. Neuropathol. Exp. Neurol. 70, 551–567 10.1097/NEN.0b013e31821f891f 21666502 PMC3118973

[B102] Shively S. Scher A. I. Perl D. P. Diaz-Arrastia R. (2012). Dementia resulting from traumatic brain injury: what is the pathology? Arch Neurol. 69, 1245–1251 22776913 10.1001/archneurol.2011.3747 PMC3716376

[B103] Sinson G. Perri B. R. Trojanowski J. Q. Flamm E. S. McIntosh T. K. (1997). Improvement of cognitive deficits and decreased cholinergic neuronal cell loss and apoptotic cell death following neurotrophin infusion after experimental traumatic brain injury. J. Neurosurg. 86, 511–518 10.3171/jns.1997.86.3.0511 9046309

[B104] Sivanandam T. M. Thakur M. K. (2012). Traumatic brain injury: a risk factor for Alzheimer's disease. Neurosci. Biobehav. Rev. 36, 1376–1381 10.1016/j.neubiorev.2012.02.013 22390915

[B105] Smith D. H. Chen X. H. Iwata A. Graham D. I. (2003a). Amyloid beta accumulation in axons after traumatic brain injury in humans. J. Neurosurg. 98, 1072–1077 10.3171/jns.2003.98.5.1072 12744368

[B106] Smith C. Graham D. I. (2003b). Tau immunohistochemistry in acute brain injury. Neuropathol. Appl. Neurobiol. 29, 496–502 10.1046/j.1365-2990.2003.00488.x 14507341

[B107] Smith D. H. Chen X. H. Nonaka M. Trojanowski J. Q. Lee V. M. Saatman K. E. (1999). Accumulation of amyloid beta and tau and the formation of neurofilament inclusions following diffuse brain injury in the pig. J. Neuropathol. Exp. Neurol. 58, 982–992 10.1097/00005072-199909000-00008 10499440 PMC13479983

[B108] Smith D. H. Chen X. H. Pierce J. E. Wolf J. A. Trojanowski J. Q. Graham D. I. (1997). Progressive atrophy and neuron death for one year following brain trauma in the rat. J. Neurotrauma 14, 715–727 10.1089/neu.1997.14.715 9383090

[B109] Smith D. H. Nakamura M. McIntosh T. K. Wang J. Rodriguez A. Chen X. H. (1998). Brain trauma induces massive hippocampal neuron death linked to a surge in beta-amyloid levels in mice overexpressing mutant amyloid precursor protein. Am. J. Pathol. 153, 1005–1010 10.1016/S0002-9440(10)65643-X 9736050 PMC1853010

[B110] Stern R. A. Riley D. O. Daneshvar D. H. Nowinski C. J. Cantu R. C. McKee A. C. (2011). Long-term consequences of repetitive brain trauma: chronic traumatic encephalopathy. PM R 3 10 Suppl 2, S460–S467 10.1016/j.pmrj.2011.08.008 22035690

[B111] Stone J. R. Okonkwo D. O. Singleton R. H. Mutlu L. K. Helm G. A. Povlishock J. T. (2002). Caspase-3-mediated cleavage of amyloid precursor protein and formation of amyloid Beta peptide in traumatic axonal injury. J. Neurotrauma 19, 601–614 10.1089/089771502753754073 12042095

[B112] Stover J. F. Schoning B. Beyer T. F. Woiciechowsky C. Unterberg A. W. (2000). Temporal profile of cerebrospinal fluid glutamate, interleukin-6, and tumor necrosis factor-alpha in relation to brain edema and contusion following controlled cortical impact injury in rats. Neurosci. Lett. 288, 25–28 10.1016/S0304-3940(00)01187-3 10869807

[B113] Sutton R. L. Lescaudron L. Stein D. G. (1993). Unilateral cortical contusion injury in the rat: vascular disruption and temporal development of cortical necrosis. J. Neurotrauma 10, 135–149 10.1089/neu.1993.10.135 8411217

[B114] Szczygielski J. Mautes A. Steudel W. I. Falkai P. Bayer T. A. Wirths O. (2005). Traumatic brain injury: cause or risk of Alzheimer's disease? A review of experimental studies. J. Neural Transm. 112, 1547–1564 10.1007/s00702-005-0326-0 15959838

[B115] Tamagno E. Guglielmotto M. Bardini P. Santoro G. Davit A. Di Simone D. (2003). Dehydroepiandrosterone reduces expression and activity of BACE in NT2 neurons exposed to oxidative stress. Neurobiol. Dis. 14, 291–301 10.1016/S0969-9961(03)00131-1 14572450

[B116] Tehranian R. Rose M. E. Vagni V. Griffith R. P. Wu S. Maits S. (2006). Transgenic mice that overexpress the anti-apoptotic Bcl-2 protein have improved histological outcome but unchanged behavioral outcome after traumatic brain injury. Brain Res. 1101, 126–135 10.1016/j.brainres.2006.05.049 16782076

[B117] Tokuda T. Ikeda S. Yanagisawa N. Ihara Y. Glenner G. G. (1991). Re-examination of ex-boxers' brains using immunohistochemistry with antibodies to amyloid beta-protein and tau protein. Acta Neuropathol. 82, 280–285 10.1007/BF00308813 1759560

[B118] Town T. (2009). Alternative Abeta immunotherapy approaches for Alzheimer's disease. CNS Neurol. Disord. Drug Targets 8, 114–127 10.2174/187152709787847306 19355932 PMC2712251

[B119] Town T. Laouar Y. Pittenger C. Mori T. Szekely C. A. Tan J. (2008). Blocking TGF-beta-Smad2/3 innate immune signaling mitigates Alzheimer-like pathology. Nat. Med. 14, 681–687 18516051 10.1038/nm1781 PMC2649699

[B120] Town T. Nikolic V. Tan J. (2005). The microglial “activation” continuum: from innate to adaptive responses. J. Neuroinflammation 2: 24 10.1186/1742-2094-2-24 16259628 PMC1298325

[B121] Tran H. T. LaFerla F. M. Holtzman D. M. Brody D. L. (2011a). Controlled cortical impact traumatic brain injury in 3xTg-AD mice causes acute intra-axonal amyloid-beta accumulation and independently accelerates the development of tau abnormalities. J. Neurosci. 31, 9513–9525 10.1523/JNEUROSCI.0858-11.2011 21715616 PMC3146343

[B122] Tran H. T. Sanchez L. Esparza T. J. Brody D. L. (2011b). Distinct temporal and anatomical distributions of amyloid-beta and tau abnormalities following controlled cortical impact in transgenic mice. PLoS ONE 6:e25475 10.1371/journal.pone.0025475 21980472 PMC3183029

[B123] Tyor W. R. Avgeropoulos N. Ohlandt G. Hogan E. L. (2002). Treatment of spinal cord impact injury in the rat with transforming growth factor-beta. J. Neurol. Sci. 200(1–2), 33–41 10.1016/S0022-510X(02)00113-2 12127673

[B124] Uryu K. Chen X. H. Martinez D. Browne K. D. Johnson V. E. Graham D. I. (2007). Multiple proteins implicated in neurodegenerative diseases accumulate in axons after brain trauma in humans. Exp. Neurol. 208, 185–192 10.1016/j.expneurol.2007.06.018 17826768 PMC3979356

[B125] Uryu K. Laurer H. McIntosh T. Pratico D. Martinez D. Leight S. (2002). Repetitive mild brain trauma accelerates Abeta deposition, lipid peroxidation, and cognitive impairment in a transgenic mouse model of Alzheimer amyloidosis. J. Neurosci. 22, 446–454 11784789 10.1523/JNEUROSCI.22-02-00446.2002 PMC6758680

[B126] Walker L. C. Callahan M. J. Bian F. Durham R. A. Roher A. E. Lipinski W. J. (2002). Exogenous induction of cerebral beta-amyloidosis in betaAPP-transgenic mice. Peptides 23, 1241–1247 10.1016/S0196-9781(02)00059-1 12128081

[B127] Weitz T. M. Town T. (2012). Microglia in Alzheimer's Disease: It's All About Context. Int. J. Alzheimers Dis. 2012: 314185 10.1155/2012/314185 22779026 PMC3388286

[B128] Yakovlev A. G. Knoblach S. M. Fan L. Fox G. B. Goodnight R. Faden A. I. (1997). Activation of CPP32-like caspases contributes to neuronal apoptosis and neurological dysfunction after traumatic brain injury. J. Neurosci. 17, 7415–7424 9295387 10.1523/JNEUROSCI.17-19-07415.1997 PMC6573442

[B129] Yatsiv I. Grigoriadis N. Simeonidou C. Stahel P. F. Schmidt O. I. Alexandrovitch A. G. (2005). Erythropoietin is neuroprotective, improves functional recovery, and reduces neuronal apoptosis and inflammation in a rodent model of experimental closed head injury. FASEB J. 19, 1701–1703 16099948 10.1096/fj.05-3907fje

[B130] Yoshiyama Y. Uryu K. Higuchi M. Longhi L. Hoover R. Fujimoto S. (2005). Enhanced neurofibrillary tangle formation, cerebral atrophy, and cognitive deficits induced by repetitive mild brain injury in a transgenic tauopathy mouse model. J. Neurotrauma 22, 1134–1141 10.1089/neu.2005.22.1134 16238489

[B131] Yu F. Zhang Y. Chuang D. M. (2012). Lithium reduces BACE1 overexpression, beta amyloid accumulation, and spatial learning deficits in mice with traumatic brain injury. J. Neurotrauma 29, 2342–2351 10.1089/neu.2012.2449 22583494 PMC3430485

[B132] Zemlan F. P. Rosenberg W. S. Luebbe P. A. Campbell T. A. Dean G. E. Weiner N. E. (1999). Quantification of axonal damage in traumatic brain injury: affinity purification and characterization of cerebrospinal fluid tau proteins. J. Neurochem. 72, 741–750 10.1046/j.1471-4159.1999.0720741.x 9930748

